# Neural simulation pipeline: Enabling container-based simulations on-premise and in public clouds

**DOI:** 10.3389/fninf.2023.1122470

**Published:** 2023-03-21

**Authors:** Karol Chlasta, Paweł Sochaczewski, Grzegorz M. Wójcik, Izabela Krejtz

**Affiliations:** ^1^Department of Computer Science, Polish-Japanese Academy of Information Technology, Warsaw, Poland; ^2^Department of Management in Networked and Digital Societies, Kozminski University, Warsaw, Poland; ^3^Department of Neuroinformatics and Biomedical Engineering, Institute of Computer Science, Maria Curie-Sklodowska University in Lublin, Lublin, Poland; ^4^Eye Tracking Research Center, SWPS University, Warsaw, Poland

**Keywords:** GENESIS, scientific workflows, Docker, computer simulations, liquid state machine (LSM)

## Abstract

In this study, we explore the simulation setup in computational neuroscience. We use GENESIS, a general purpose simulation engine for sub-cellular components and biochemical reactions, realistic neuron models, large neural networks, and system-level models. GENESIS supports developing and running computer simulations but leaves a gap for setting up today's larger and more complex models. The field of realistic models of brain networks has overgrown the simplicity of earliest models. The challenges include managing the complexity of software dependencies and various models, setting up model parameter values, storing the input parameters alongside the results, and providing execution statistics. Moreover, in the high performance computing (HPC) context, public cloud resources are becoming an alternative to the expensive on-premises clusters. We present Neural Simulation Pipeline (NSP), which facilitates the large-scale computer simulations and their deployment to multiple computing infrastructures using the infrastructure as the code (IaC) containerization approach. The authors demonstrate the effectiveness of NSP in a pattern recognition task programmed with GENESIS, through a custom-built visual system, called RetNet(8 × 5,1) that uses biologically plausible Hodgkin–Huxley spiking neurons. We evaluate the pipeline by performing 54 simulations executed on-premise, at the Hasso Plattner Institute's (HPI) Future Service-Oriented Computing (SOC) Lab, and through the Amazon Web Services (AWS), the biggest public cloud service provider in the world. We report on the non-containerized and containerized execution with Docker, as well as present the cost per simulation in AWS. The results show that our neural simulation pipeline can reduce entry barriers to neural simulations, making them more practical and cost-effective.

## 1. Introduction

Neural simulation is a computational approach that involves building and running computer models of the structure and function of the brain or parts of the brain. It can be used to study the brain and how it works as well as to explore and test hypotheses about brain function in health and disease. Using neural simulation can be useful in studying and understanding the complexity of certain central nervous system (CNS) disorders as it allows researchers to investigate and analyze the brain's structure and function in a controlled and precise manner (Eliasmith and Trujillo, 2014). This can help identify potential targets for therapeutic intervention and test the effects of different treatments or interventions on the brain function.

Neural simulation can be used to study the neural basis of disorders such as Alzheimer's disease or autism spectrum disorder as well as to explore the mechanisms underlying brain development and plasticity (Duch, 2000). The computer simulation can also be used to investigate the effects of drugs and other interventions on the brain function and to identify potential therapeutic targets for the treatment of CNS disorders, e.g., using different boundary conditions (Gholampour and Fatouraee, 2021).

Alzheimer's disease is a progressive brain disorder that causes problems with memory, thinking, and behavior. It is the most common cause of 60–70% of cases of progressive cognitive impairment in older adults, and it is estimated to affect at least 2.3 million (ranging from 1.09 to 4.8 million) people in the United States (Cummings and Cole, 2002). The prevalence of Alzheimer's disease increases with age, and it is estimated that ~1 in 10 people over the age of 65 and nearly half of those over the age of 85 have the disease (Weuve et al., 2015).

Autism spectrum disorder is a neurodevelopmental disorder characterized by difficulties with social interaction and communication, as well as repetitive behaviors and interests (Hirota and King, 2023). It is estimated to affect 2.3% of children aged 8 years and ~2.2% of adults in the United States, with boys being four times more likely to be diagnosed with ASD than girls. The prevalence of ASD has increased significantly over the past few decades; however, it is not clear whether this is due to an actual increase in the number of cases or due to improved detection and diagnosis (Landrigan, 2010).

Large-scale simulations of biologically realistic neural networks often require expensive computational resources (Markram, 2006; Eliasmith and Trujillo, 2014). They also create challenges with storing the massive amounts of their data (Eliasmith and Trujillo, 2014) or with developing, distributing, and maintaining their codebase (Davison et al., 2009). Their configuration and model deployment might present a significant barrier for many researchers tackling biocybernetic modeling. This is due to both methodological and IT challenges (Eliasmith and Trujillo, 2014), which include maintaining software dependencies and executing on different hardware infrastructures. The task is not trivial from a technical perspective even when using as well-established simulation engine as GENESIS (Crone et al., 2019).

We focus on GENESIS (Bower and Beeman, 1998), the most cited simulation engine (Tikidji-Hamburyan et al., 2017), originally developed in 1989 by Dr. James M. Bower in his laboratory at the California Institute of Technology (Caltech). GENESIS is designed to be easily extensible and adaptable to run on different HPC clusters. Apart from its maturity, one of the key advantages of using GENESIS is its openness. The software is distributed under GNU General Public License (GNU GPL; License, 1989), so its users have the freedom to run, share, and modify the simulation engine's source code, and any derivative work must also be distributed under the same or equivalent license terms. There are two major flavors of the simulation engine: a standard GENESIS and a PGENESIS that runs on a wide range of hardware using the Message Passing Interface (MPI) or the Parallel Virtual Machine (PVM; Bower, 2000).

There were several attempts to use the computational approach to tackle Alzheimer's disease (Duch, 2000; Chlasta and Wołk, 2021) or autism spectrum disorder (ASD; Duch et al., 2012, 2013; Dobosz et al., 2013; Duch, 2019). Our project aims to deliver new tools facilitating the study of brain function through software containerization based on Docker and can be used to inform the development of new therapies and interventions for a variety of CNS disorders, including Alzheimer's disease and autism spectrum disorder. However, the anticipated advance goes beyond just brain network simulations, and in our view, it also includes computational neuropharmacology (Aradi and Érdi, 2006) as well as increasingly computational psychology based on “neuron-like” processing principles, complementing its “traditional” computational neuroscience background (O'reilly and Munakata, 2000).

### 1.1. Project idea

**Figure 1:**
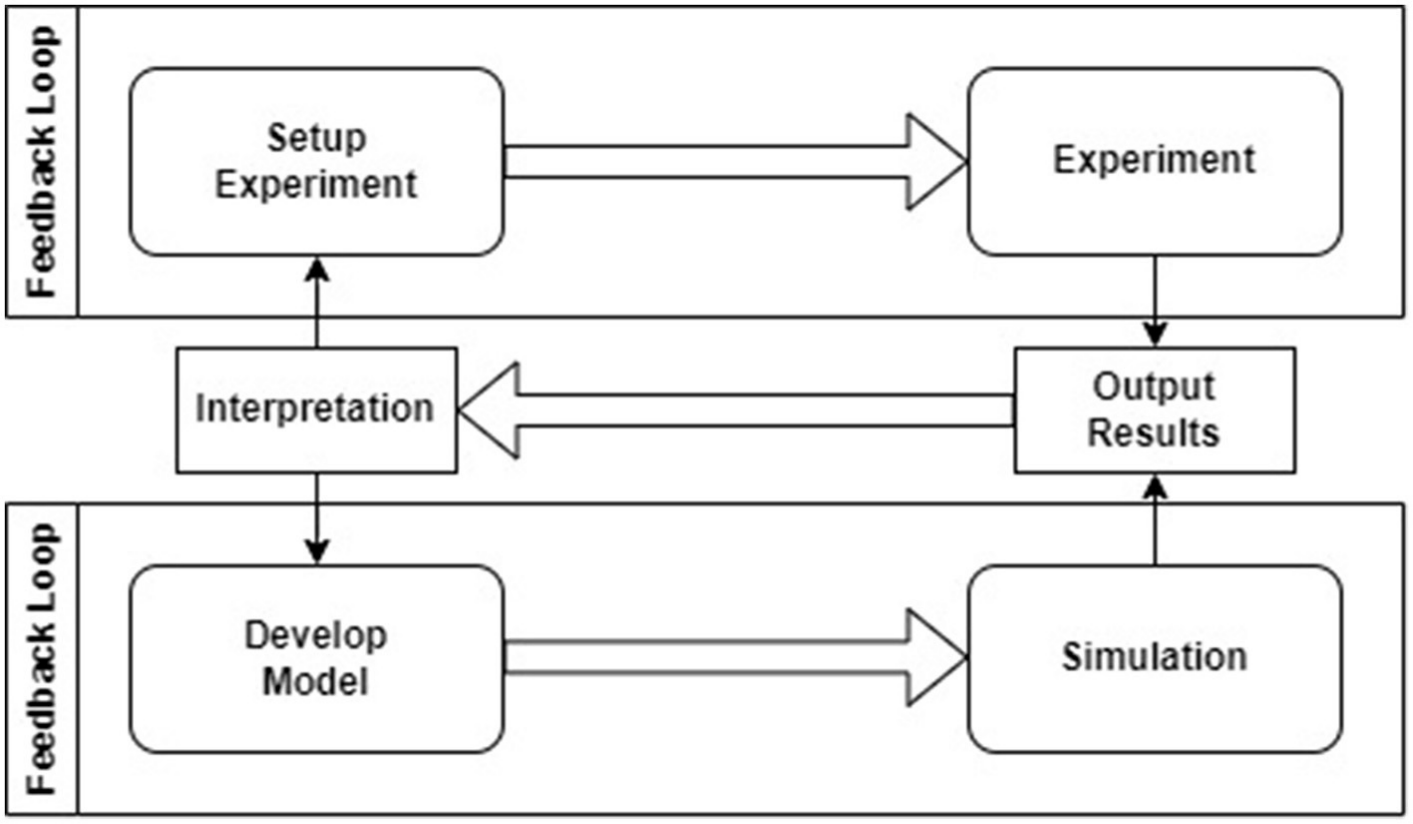
The relationship between experiment and simulation in computational neuroscience based on GENESIS documentation (Bower et al., 2003).

Authors claim that numerical simulations must integrate a robust model development methodology, with adequate testing and simulation steering workflows to increase scientific throughput and improve utilization of current and next-generation computational infrastructure, available both on-premise and in-cloud. To this end, there is the need to transform the end-to-end computational experiment workflow from one that is non-universal and manual to one that is standardized and automated. Figure 1 presents the relationship between the simulation setup and simulation run in computational neuroscience. As it can be seen, *conducting experiment* and *running simulation* are two distinct iterative loops connected by a feedback process. This process uses the *interpretation of output results* to design *new simulation setups* and develop *new cybernetic models*.

GENESIS (Bower and Beeman, 1998) supports the lower loop within the system as shown above, but it leaves *a gap for setting up and executing the simulations* (e.g., setting up model parameter values, different stimuli, storing the parameters, and providing execution statistics). A similar gap was identified for other popular simulation engines (Tikidji-Hamburyan et al., 2017) like BRIAN (Goodman and Brette, 2008), NEST (Gewaltig and Diesmann, 2007), and NEURON (Hines and Carnevale, 2001) or the most popular functional simulation engine called Nengo (Bekolay et al., 2014). Moreover, each simulator uses its own programming or configuration language, what leads to challenges in porting models from one simulation engine to another and managing them (Davison et al., 2009). These problems triggered the idea of creating a more universal simulation pipeline called *Neural Simulation Pipeline* (NSP).

NSP manages simulations and allows them to be saved and defined for different simulation engines in a unified way. The framework provides both local and remote queues for executing simulations. These queues can be executed regardless of the hardware platform through Docker containers running in the cloud or on-premise. The NSP also enables a faster analysis of experimental data. This is because (1) all the simulation results are stored centrally using a single storage service, (2) they can be managed using a set of standard NSP scripts on both Linux and Windows machines, (3) or through the reusable NSP variables, and (4) all the experimental data gets partially pre-processed, by aggregating the results together with the statistics on the execution environment (e.g., simulation run-times, detailed information about CPUs, memory, and operating system processes, and simulation engine specific statistics).

## 2. Materials and methods

### 2.1. GENESIS simulation engine

Brain network simulations can be performed with a GENESIS simulation engine (Bower et al., 2003). GENESIS (Goddard and Hood, 1997; Bower and Beeman, 2012) is an object-oriented multi-function neural simulation software package that allows scientists to flexibly build high-fidelity neurobiological models. These models are capable of simulating brain functions on different levels from the level of small sub-cellular components to sophisticated large and complex neural networks.

Moreover, GENESIS from version 2.3 contains Kinetikit, an interface and utilities for developing simulations of chemical kinetics. This extension contains a comprehensive graphical simulation environment for modeling biochemical signaling pathways using deterministic and stochastic methods (Vayttaden and Bhalla, 2004). The extended GENESIS becomes a tool to investigate the biomechanics of the brain including its time-dependent temperature and pressure variations, or the liquid behaviors in contrast to ideal conditions. As such, the GENESIS/Kinetikit simulations could be used to study the dynamics of cerebrospinal fluid flow and pressure, which can provide valuable information for diagnosing and managing fluid disorders or testing the effects of different interventions or optimizing treatment strategies (Musilova and Sedlar, 2021). In authors' view, this justifies the positioning of our article to the Frontiers' Research Topic “Modeling and Simulation of Cerebrospinal Fluid Disorders.”

GENESIS simulations are programmed using objects that have inputs on which mathematical operations are performed and then, based on the result of those operations, generate outputs which become inputs to other objects. Neurons in GENESIS models are built from these basic components in a *compartmental fashion* (Beeman, 2005) using a GENESIS Script Language Interpreter (SLI) that provides the programmer with a built-in language to define and manipulate these GENESIS objects. In the compartmental approach, neuron's compartments in GENESIS are linked to their ion channels, and the channels are linked together to form multi-compartmental neurons of up to 50–74 compartments per neuron. GENESIS simulations scale on super-computing resources to neural network sizes as large as 9 × 106 neurons with 18 × 109 synapses and 2.2 × 106 neurons with 45 × 109 synapses (Crone et al., 2019).

### 2.2. Containerization with Docker

Authors believe that the problem of developing, testing, and deploying new simulation setups, as well as their different software dependencies could be resolved using a container platform like Docker (Merkel, 2014). According to the recent IDC's white paper (Chen, 2018), Docker is the most popular container platform. Software containerization makes it possible to use provider-agnostic computing (IaC) in the way that the required resources can be specified in a simple configuration file for multiple deployments to different hardware architectures (Naik, 2022).

As summarized by Nickoloff and Kuenzli (2019), the Docker platform uses the low-level operating system kernel internals to run applications in containers using the *Docker Engine*. The architecture of Docker containers relies on both *namespaces* and *control groups*. The process is transparent for the applications as a container is a ring-fenced area of the operating system with limits imposing on how much system resource it can use. The engine creates a layer of abstraction for all the required kernel internals and creates a container that is designed for hosting specific applications and their dependencies (Merkel, 2014). Although the containers can be deployed and managed manually, most organizations automate the processes using pipelines (Al Jawarneh et al., 2019).

In spite of a wide enterprise adoption, there are significant problems with resource allocations (de Bayser and Cerqueira, 2017) when using Docker containers on the HPC platform and running simulations using MPI communications with an SLURM scheduler (Yoo et al., 2003), a popular combination of tools used for large scale simulations. These problems are resolved using additional front-ends allocating the containers, or developing the alternative containerization systems (Azab, 2017).

In this article, we present a simple alternative, the *Neural Simulation Pipeline* (NSP), that is developed by Bash (Ramey, 1994) and PowerShell (Holmes, 2012) and does not require SLURM to execute simulations.

### 2.3. Simulation setting

We evaluate NSP through executing simulations in the Amazon Web Services (AWS) cloud environment and on-premise at the Hasso Plattner Institute. We selected AWS because in the last 3 years, it has remained the biggest Infrastructure as a Service (IaaS) public cloud provider in the world if measured by both reported revenue and market share. The company achieved a revenue of $35.4 billion and a market share of 38.9% last year. They were followed by Microsoft, Alibaba, Google, and Huawei, collectively amounting to the 80% of the cloud computing market globally last year (Gartner, 2022). These numbers are significant because AWS is the biggest vendor and can deliver vast benefits of economies of scale, while as the report suggests “cloud-native becomes the primary architecture for any modern computing workloads.” In the our view, this should and will affect the way large scale computer simulations are executed in future. There might be no return to the large and expensive HPC projects like the Blue Brain Project (Markram, 2006), that simulated a single neural column of 10,000 neurons using 8,000 cores of the IBM Blue Gene supercomputer (that is, 1.25 neuron per core).

All the services that allowed us to perform the containerized execution of NSP in AWS are presented in **Figure 4** and documented on AWS Cloud Products website.^1^ These are Amazon Elastic Container Service (ECS), Amazon Elastic Compute Cloud (ECC), and Amazon Elastic Load Balancing (ELB), whose task definitions were used by Amazon ECS Cluster, AWS Secrets Manager, AWS CodePipeline, AWS CodeBuild, AWS CodeDeploy, Amazon Elastic Container Registry (ECR), Amazon CloudWatch, AWS Simple Cloud Storage (S3), AWS Identity and Access Management (IAM), Amazon Virtual Private Cloud (VPC), and Amazon Route 53 (R53). All these services were used to provision a physical Intel(R) Xeon(R) CPU E5-2686 v4 @ 2.30 GHz with 14 GB RAM that run each container using a task configured to use 1 CPU (*task*_*cpu* = 1, 024) and 8 GB of RAM (*task*_*memory* = 8, 192). To summarize, we executed our simulations on Amazon Elastic Compute Cloud (machines), using Amazon Elastic Container Service (Docker) through Amazon Elastic Load Balancing (load balancing) and Amazon Elastic Container Registry (Docker registry) in Amazon Virtual Private Cloud (networking).

We managed all our AWS services through the Infrastructure as Code approach (Kumar et al., 2023) with Terraform v1.0.11. As a result, the whole configuration of our cloud environment is stored as Terraform code in the main NSP repository (*nsp-code*) under infra\core-infra sub-folder for the VPC configuration, and under infra\nsp-lb-service for all the other associated services. This configuration can be used as a reference point for any future deployments of NSP by the members of scientific community.

We also received access to the machines from Hasso Plattner Institute, allowing us to compile and install all the required software, e.g., the latest version of the GENESIS simulation environment (Bower et al., 2003) (version 2.4 from May 2019) and Docker (Merkel, 2014) platform (version 20.10.08 from July 2021). The machine we received was equipped with 8 CPUs: 8 x 2.00 GHz, memory (RAM): 7.79 GB. We used the local Docker Registry at HPI at registry.fsoc.hpi.uni-potsdam.de in the development process. The other computing resource that is used in this project is the Ubuntu 18.04.5 bionic virtual machine (vm-20211005- 001.fsoc.hpi.uni-potsdam.de, running Linux 4.15.0-143-generic on x86_64 architecture). It was used as our development machine and on-premise execution environment.

### 2.4. Architecture of Neural Simulation Pipeline

Different components of NSP connect to the AWS cloud *via* the AWS CLI (AWS Command Line Interface), sending requests to the AWS services by using HTTPS on TCP port 443. For security reasons, we propose to create at least two types of user accounts with AWS IAM service: an account for *a user persona*, focused on simulation design and execution (the data), and a separate one for *a developer and maintainer persona*, focused on the development of simulation models and administration of the pipeline through the code. We propose that these two different types of personas interact with the system *via* different interfaces:

- *A user persona, via NSP scripts*, as described in Table 1.
- *A developer and maintainer persona, via* Git client (code), AWS CLI, and AWS Management Console (actions; see text footnote 1).

**Table 1:** Full list of scripts in Neural Simulation Pipeline.

**Component**	**User scripts**	**Container scripts**
Simulation	buildNspImage.sh	configAWSCLI.sh
preparation	listModels.sh (.ps1)	validatePositive Integer.sh
	loadModels.sh (.ps1)	validateReal Number.sh
	pullNspImage.sh (.ps1)	validateRange.sh
	pushNspImage.sh	
	runUnitTest.sh (.ps1)	
	runUnitTestCheck.sh	
	startNspContainer.sh (.ps1)	
	getCredentials.ps1	
Simulation	loginNspContainer.sh (.ps1)	runSim.sh
execution	runSimLocally.sh (.ps1)	runSimLocally.sh
	runSimRemotely.sh (.ps1)	runSimManagerS3.sh
	runSampleSim.sh (.ps1)	showStat.sh
	showNspQueue.sh (.ps1)	showSystemInfo.sh
		calculatePeriod.sh
		writeDebug.sh
		writeOutput.sh
Simulation	downloadSimS3.sh (ps1)	downloadSim.sh
post-processing	listSim.sh (ps1)	downloadModel.sh
	showNspContainerLogs.sh (.ps1)	listSim.sh
	stopNspContainer.sh (.ps1)	saveStat.sh
	deleteNspImages.sh (.ps1)	

**Figure 2:**
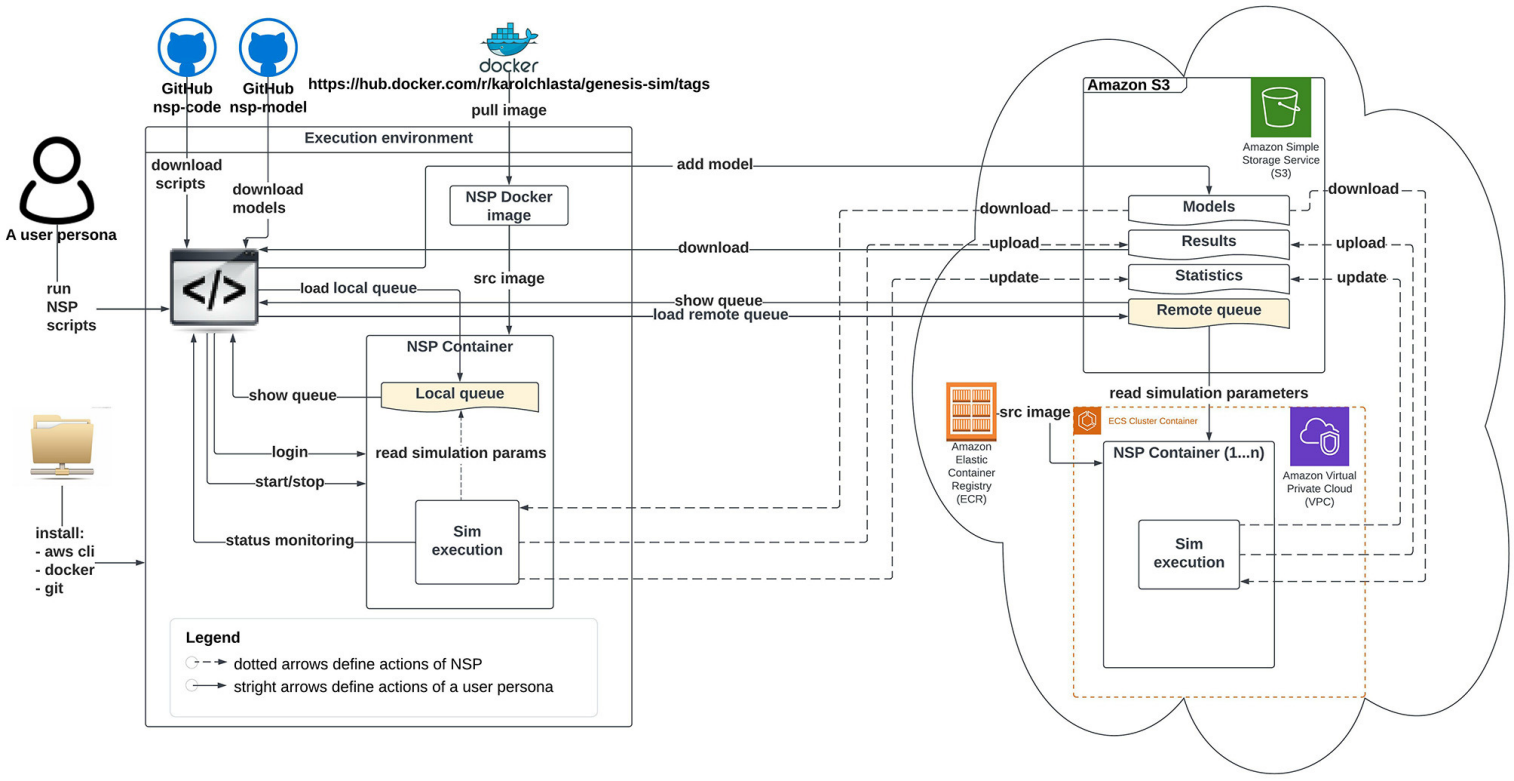
Schematic diagram of neural simulation pipeline showing a user workflow. This persona interacts with the system *via* NSP scripts.

**Figure 3:**
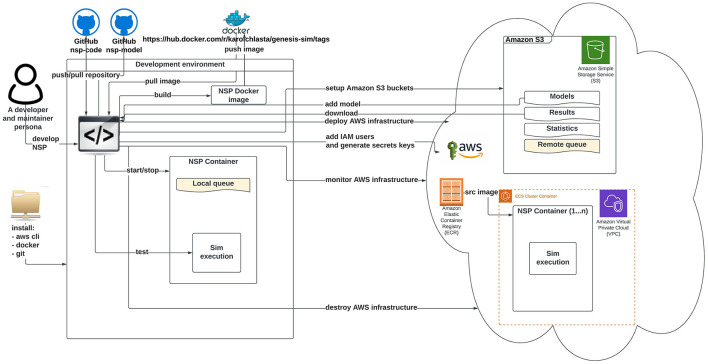
Schematic diagram of neural simulation pipeline showing a developer and maintainer workflow. This persona interacts with the system *via* Git client (code), AWS CLI, and AWS Management Console.

Figures 2, 3 illustrate an architectural blueprint of neural simulations pipeline. They explain how different elements of the pipeline run and interact with the two categories of the users. The first, as shown in Figure 2, focuses on a user workflow, whereas Figure 3 highlights a developer and maintainer workflow, explaining what the key actions are and if they are performed by the users or by the pipeline itself.

A user workflow in Figure 2 presents all the key user interactions with the NSP through solid lines and straight arrows. These key actions are installing the software pre-requisites, downloading our NSP scripts, pulling our NSP Docker image from the DockerHub registry, starting a local container, defining a local or remote simulation queue, as well as monitoring the status of execution, and finally downloading the simulation results. After all the local simulations are finished, it is a good practice to stop the container to release system resources. The user workflow is supported by NSP user scripts described individually in Appendix (Section 1).

However, if a remote cloud-based execution is attempted, the NSP Docker image from the DockerHub is not needed. The pipeline provides the automated build and management of NSP image through a native Amazon ECR service, that guarantees the optimal performance and connectivity to other AWS services. All the repetitive actions related to data movement to/from a NSP container have been automated through NSP container scripts, described individually in Appendix (Section 2); Figure 2 shows these actions with dotted lines. The figure also presents two simulations queues available in NSP (*localSimulationQueue.nsp* or *remoteSimulationQueue.nsp*, in yellow), with the local queue being active since the start of local container, and the remote queue being checked for the simulation tasks in a definable interval.

Figure 3 shows key actions performed in a developer and maintainer workflow. The persona configures and operates the cloud-based execution environment. Our AWS NSP infrastructure is created on-demand *via* the Terraform (Brikman, 2016) using IaC approach. The Terraform configuration covers all the required services including network components of VPC, Amazon Elastic Container Service cluster setup, and the configuration of AWS CodePipeline using AWS CodeBuild and AWS CodeDeploy. The AWS CodePipeline task is triggered automatically by a commit done to the “main” branch of the *nsp-code* repository. The AWS CodePipeline downloads the *nsp-code* repository from the of “main” branch and runs the AWS build task that executes Docker commands from the Dockerfile defined in the repository. As a result, the new NSP Docker image is created and pushed into the Amazon Elastic Container registry.

**Figure 4:**
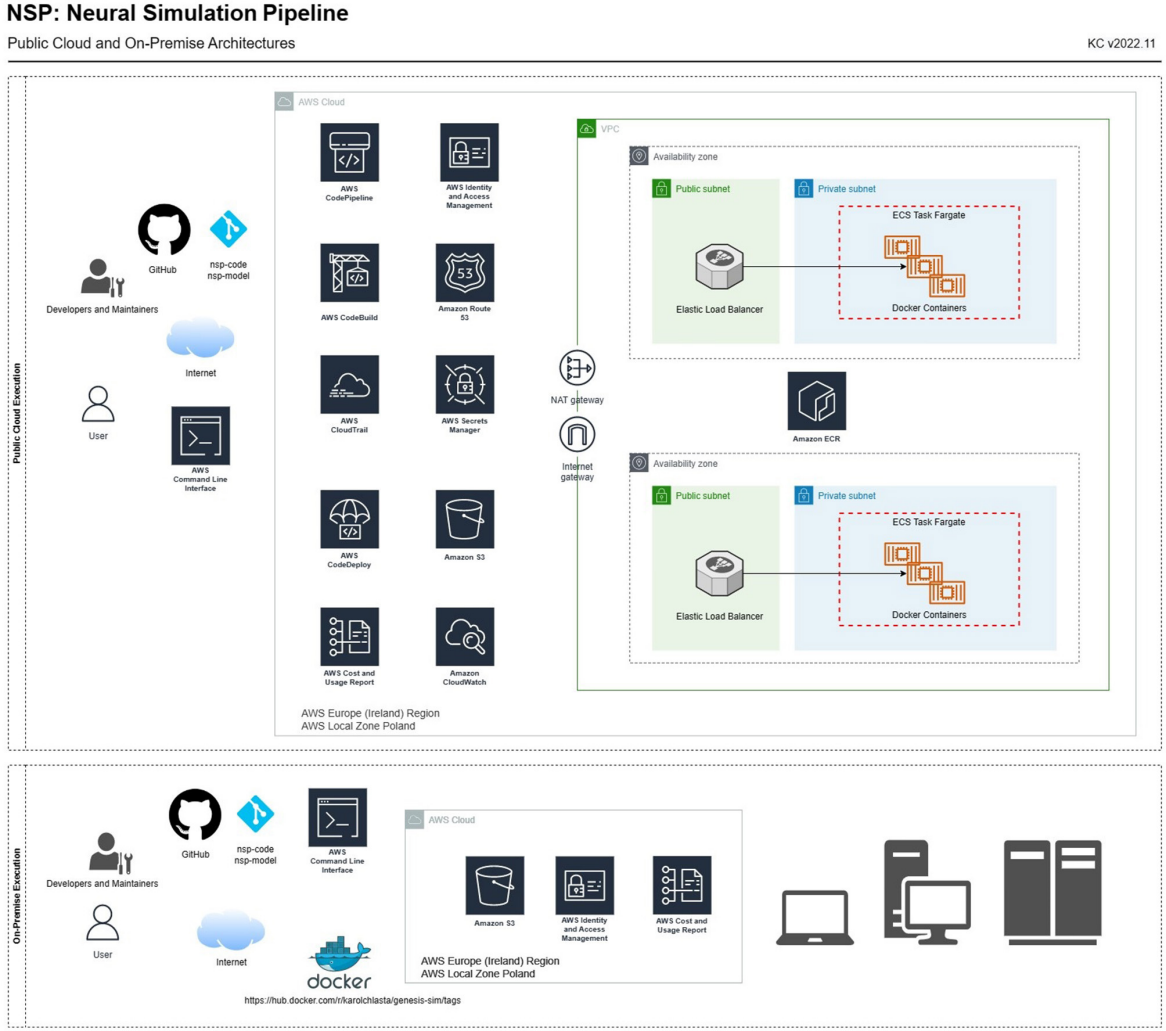
NSP architecture for a full public cloud (AWS) simulation execution **(top)** and an on-premise execution **(bottom)**. The diagram presents both AWS services and network components needed to set up a VPC required to execute simulations in-cloud through Amazon Elastic Container Registry and Amazon Elastic Container Cluster running on AWS Fargate, a serverless, pay-as-you-go compute engine.

Figure 4 complements the architectural overview with a list of services needed to execute the simulations either (1) through the public cloud (AWS) or (2) using on-premise infrastructure. In both use cases, we adopted a central storage service (Amazon S3), whose storage infrastructure holds those as follows:

- The simulation data (understood as both the input parameters and the results of simulations).
- The model's source code for the reference of the exact version of the simulation to the results.
- The task queue for simulations, that are executed by Docker containers.
- Supplemental experimental data including environment statistics and cost reports.

Each type of execution requires provisioning a different set of services:

1. *Cloud execution* (upper part of Figure 4). In this use case, we use standard AWS services, allowing for an automated build of the Docker image (based on the *Dockerfile*, defining the compilation and installation steps of all the necessary libraries, and software components; file available in the main *nsp-code* repository). It provides AWS CodePipeline, AWS CodeBuild, and CodeDeploy, that produces the container image available in Amazon Elastic Container Registry. The container image is maintained through the Amazon ECR service, with individual containers being managed through the AWS Fargate engine. All the logs from simulation processing, as well as from the containers and the pipeline, are stored in AWS CloudWatch. After NSP is configured in the AWS cloud, the simulations can be run through AWS containers using the remote simulation queue, that is defined as a text file at *s3://nsp-project/requests/remoteSimulationQueue.nsp*. This queue, when populated with a list of simulations, will be executed by AWS containers.
2. *On-premise execution* (lower part of Figure 4). In this use case, we execute simulations on own, or shared computer such as a laptop, workstation, or a computational cluster. As a result, a local Docker client needs to be installed, and a public DockerHub service can be used to download our NSP image^2^ containing the latest, pre-configured version of GENESIS simulation engine.

There are three functional components of NSP: (1) simulation preparation, (2) simulation execution, and (3) simulation post-processing. The preparation component manages the simulation's input data into a format suitable for the simulation, while the execution module performs the actual simulation execution using a selected engine. The current version of NSP also allows to select either the standard or parallel version of the GENESIS simulation engine. The selection is performed *via* a parameter of *runSim.sh* script; with value *parallelMode = 1* indicating a parallel run. Finally, the post-processing module facilitates the analysis of output data and generates the final results for a given simulation.

These components are built around two types of scripts. These are the *container scripts*, automating the simulation tasks within the application container, and the *user scripts*, responsible for the interaction with pipeline's end-user. Both types of script are summarized in Table 1 and described in Appendix (Section 1) (user scripts) and Appendix (Section 2) (container scripts). All the NSP scripts are installed automatically with our Docker image.

The NSP facilitates an automated testing of the simulation code (models) through *runUnitTest.sh* and *runUnitTestCheck.sh*. These files contain sample tests. If a new model is developed, then the new test scripts might need to be created in an analogous way. Ideally, the model will have full test coverage, that gives confidence that a given model is tested, and any bug is identified early in the development process. Applying this best practice is especially important for the long running brain simulations, whose bugs could often only be identified *post-hoc*, e.g., after running for several days (or weeks) on expensive supercomputers. These are the three scripts to facilitate parameter validation: *validateRange.sh, validatePositiveInteger.sh*, and *validateRealNumber.sh)*. There are also other scripts supporting the simulation setup and execution. All the 35 Bash and 16 PowerShell scripts are listed in Table 1 and described individually in Appendix (Sections 1, 2).

The pipeline also allows to define and reuse certain variables that are universal and independent from the simulation engine. We call them *NSP variables*. These variables should be added to the model's source code between the special character of “$ $” (e.g., “$nspVariableName$”). As a result, the models' code can be more standardized, even across different simulation engines. Moreover, new possibilities could be created. For investigating boundary conditions to improve brain simulation process similar to what was proposed in Gholampour and Fatouraee (2021). The current version of the pipeline recognizes twelve NSP variables:

1. $modelName$
2. $simSuffix$
3. $simDesc$
4. $simTimeStepInSec$
5. $simTime$
6. $columnDepth$
7. $synapticProbability$
8. $retX$
9. $retY$
10. $parallelMode$
11. $numNodes$
12. $modelInput$

There are two types of statistics managed by the pipeline automatically through the *showSystemInfo.sh* NSP script, generating the aggregated *simulationInfo.out* per simulation. They are as follows:

- *Operating system-level statistics*. They describe the execution environment including process timings. These are generated using parameterized Linux commands of date, uname, lshw, lscpu, lsblk, df, and lspci adn smem. The script also uses *calculatePeriod.sh* subscript to calculate the exact time of simulation.
- *Simulation engine specific statistics*. They are triggered by the NSP through the GENESIS *showstat* routine.^3^

The pipeline's source code is stored in the *nsp-code* repository available publicly at GitHub.^4^ This is the main application repository used for all the container builds, and it contains the *Dockerfile* describing the automated build process for GENESIS (in nsp-server/Dockerfile). The other repository used in the project is called the *nsp-model*.^5^ It stores the source code of all the RetNet models used in our simulations.

The pipeline's configuration is managed on different levels. The local Docker containers are configured through the *config.nsp* file, while the remote AWS containers are configured *via* Terraform configuration file (modules\ecs-service\variables.tf). The minimum required configuration includes the AWS access and secrets keys for authentication as well as the basic metadata about the project including scientist's name, surname, and email. This information is automatically added to the simulation results. One of the useful NSP configuration parameters is a debug mode flag, enabled *via nsp_debug* parameter.

To summarize, we have built our NSP image for the GENESIS simulator using the official Canonical Ubuntu bionic (version bionic-2022101) from DockerHub.^6^ The automated build process installs csh, g++, libxt-dev, libxt6, libxtst6, libxtst-dev, libxmu-dev, mpich, gcc, bison, flex, libncurses5-dev, and libxt-dev. As a result, both GENESIS and its parallel version PGENESIS are compiled with all the dependencies, and our official, publicly available NSP image can be found in DockerHub. The image uses 424.83 MB and can be pulled from the DockerHub with the below command:

docker pull karolchlasta/genesis-sim:prod

We welcome new pushes of the updated NSP image with a “test” tag to DockerHub,^7^ so that thay can go through our review process and can be made available to the other members of scientific community to facilitate their simulations.

### 2.5. Simulating visual system task

Liquid state machines (Maass, 2011) are important in brain modeling and increasingly important in different engineering (Wang et al., 2022) or real-life applications (Deckers et al., 2022). The spiking neural networks built of Hodgkin–Huxley (HH) (Hodgkin and Huxley, 1952) neurons behave like liquid state machines (LSM) (Wojcik, 2012; Kamiński and Wójcik, 2015). Our Hodgkin–Huxley Liquid State Machine (HHLSM) model uses high fidelity multi-compartmental neurons with voltage-activated sodium and potassium channels. The LSM-based model of visual systems used to benchmark simulations performed with NSP had already been presented in Chlasta and Wojcik (2021). That version of the bio-inspired model was built using 4,880 Hodgkin–Huxley neurons with two main components: an *Input* (acting as a retina of the system) and *Liquid* (acting as a visual cortex, built of a single LSM column).

This research study focuses on the much larger models, with a progressively larger liquid column. The structure of each column in the model is the same, but the size has been adjusted through the NSP variable $columnDepth$ and built in six versions:

1. RetNet(8 × 5,1,25) with 1,040 neural cells placed in a rectangular cuboid of 8 × 5 × 25.
2. RetNet(8 × 5,1,50) with 2,040 neural cells placed in a rectangular cuboid of 8 × 5 × 50.
3. RetNet(8 × 5,1,75) with 3,040 neural cells placed in a rectangular cuboid of 8 × 5 × 75.
4. RetNet(8 × 5,1,100) with 4,040 neural cells placed in a rectangular cuboid of 8 × 5 × 100.
5. RetNet(8 × 5,1,200) with 8,040 neural cells placed in a rectangular cuboid of 8 × 5 × 200.
6. RetNet(8 × 5,1,300) with 12,040 neural cells placed in a rectangular cuboid of 8 × 5 × 300.

In the simulated task, we used NSP to provide each model with three different stimulus patterns of “0,” “A,” “1” (through different values of NSP $modelInput$ variable). This gave us the opportunity to evaluate the LSM system built with an increasing number of neurons in a standardized way. We simulated 1 s of this biological system (using the NSP variable $simulationTime$) across different execution environments.

## 3. Results

This article presents NSP, a simple scientific workflow management system, based on a set of 35 Bash and 16 PowerShell scripts, that manages simulations and facilitates defining and executing them across different simulation engines and execution environments in a unified way. The authors managed to validate NSP by running it in three different types of run-time environments (1) using containers in the AWS cloud, on-premise (2) on an HPI infrastructure, and (3) directly on the operating system without containerization. This simple scientific workflow system has also successfully managed the simulation queue, unified key experimental variables, collected data and experimental statistics, as well as provided basic validation of experimental parameters, monitored simulation execution, supported simulation code testing, and checking for the completeness of the simulation results.

In order to evaluate the NSP, we have performed several full-experimental cycles and shown that our LSM models react differently to three different input patterns that are numbers (0, 1) and letter (A). We performed a total of 54 simulations on the RetNet models on which we report. We measured the model execution time (CPU time), memory consumption, as well as the number of spikes in each simulation run. The exact results of these simulations are presented in Tables 2, 3. All the results and accompanying statistics have been gathered through running NSP scripts throughout H2 2022.

**Table 2:** Neural Simulation Pipeline—standard vs. containerized execution on HPI infrastructure.

**On-Premise HPI**					
**Model**	**Neurons**	**Pattern**	**Spikes**	**Execution [s]**	**Memory [MB]**
RetNet(8 × 5,1,25)	1,040	0	20,829	132.27	18.78
		A	20,827	136.13	37.97
		1	20,818	124.69	21.25
RetNet(8 × 5,1,50)	2,040	0	61,191	239.34	44.56
		A	61,236	205.75	52.51
		1	61,234	231.47	49.77
RetNet(8 × 5,1,75)	3,040	0	121,605	628.24	53.28
		A	121,646	536.32	65.27
		1	121,627	648.69	53.42
RetNet(8 × 5,1,100)	4,040	0	202,045	1269.55	88.81
		A	202,036	1269.12	94.99
		1	202,056	1321.30	72.24
RetNet(8 × 5,1,200)	8,040	0	6,512,454	34082.88	1766.86
		A	6,512,440	34065.61	1791.56
		1	6,568,685	26548.51	1760.26
RetNet(8 × 5,1,300)	12,040	0	14,568,366	102669.1	17482.09
		A	14,568,498	97643.23	17582.11
		1	14,568,473	101909.68	17169.67
**Containerized HPI**					
RetNet(8 × 5,1,25)	1,040	0	20,834	45.66	26.37
		A	114,427	185.05	46.51
		1	114,403	214.94	71
RetNet(8 × 5,1,50)	2,040	0	61,215	150.40	57.97
		A	428,368	1679.33	150.17
		1	428,500	1659.42	146.53
RetNet(8 × 5,1,75)	3,040	0	121,659	495.38	63.01
		A	942,366	3949.42	292.29
		1	942,452	3957.19	80.70
RetNet(8 × 5,1,100)	4,040	0	202,014	938.60	73.91
		A	1,656,397	7253.15	139.56
		1	1,656,444	7467.51	437.87
RetNet(8 × 5,1,200)	8,040	0	6,512,411	33000.14	1766.57
		A	6,512,452	33434.70	1766.35
		1	6,512,401	33198.74	437.87
RetNet(8 × 5,1,300)	12,040	0	14,568,503	87163.24	4745.92
		A	14,568,712	86226.93	4810.72
		1	14,457,211	87213.56	4756.54

**Table 3:** Neural Simulation Pipeline—containerized execution on AWS cloud computing infrastructure.

**Containerized AWS**						
**Model**	**Neurons**	**Pattern**	**Spikes**	**Execution [s]**	**Memory [MB]**	**Cost [USD]**
RetNet (8 × 5,1,25)	1,040	0	114,431	253.14	67.84	0.02
		A	114,425	253.53	46.07	0.02
		1	114,407	89.81	67.24	0.01
RetNet (8 × 5,1,50)	2,040	0	428,380	427.58	128.9	0.10
		A	428,390	998.58	138.48	0.10
		1	428,454	980.13	130.53	0.09
RetNet (8 × 5,1,75)	3,040	0	942,445	1879.22	280.51	0.22
		A	942,371	2033.78	291.59	0.19
		1	942,416	1737.16	273.31	0.17
RetNet (8 × 5,1,100)	4,040	0	1,656,346	3749.97	443.96	0.40
		A	1,656,455	4104.38	436.08	0.39
		1	1,656,388	3802.45	421.46	0.36
RetNet (8 × 5,1,200)	8,040	0	6,512,417	16913.23	1788.44	1.62
		A	6,512,400	16564.92	1757.01	1.58
		1	6,512,453	16850.31	1818.64	1.61
RetNet (8 × 5,1,300)	12,040	0	14,568,482	41814.32	17592.19	4.00
		A	14,568,446	41916.18	17592.21	4.01
		1	14,568,446	41824.63	17179.87	4.00

**Figure 5:**
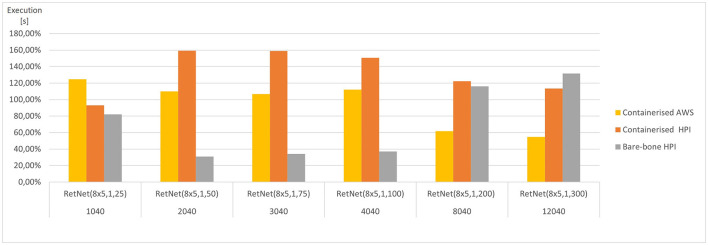
Average execution (CPU time) for each RetNet model (ranging in complexity from 1,040 to 12,040 Hodgkin–Huxley neurons) viewing three input patterns (0, A, 1), expressed as a percentage of average CPU time across all execution environments for each RetNet model. Based on Tables 2, 3.

**Figure 6:**
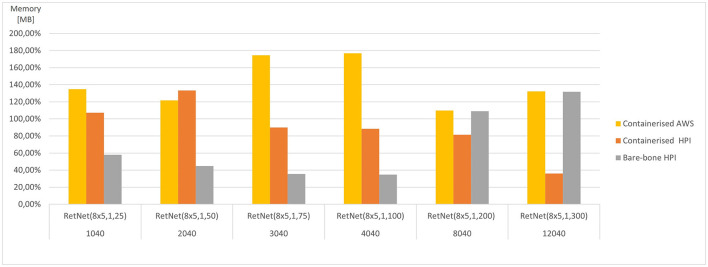
Average memory consumed for each RetNet model (ranging in complexity from 1,040 to 12,040 Hodgkin–Huxley neurons) viewing three input patterns (0, A, 1), expressed as a percentage of average memory utilization across all execution environments for each RetNet model, based on Tables 2, 3.

These aggregated, average results are presented in Figures 5, 6. They vary significantly, depending on the model complexity (number of HH neurons) and the execution environment; hence, they were averaged per model. As a result, the figures present how each execution environment performs against that average. The simulation execution time (as measured by CPU time in seconds) varies from 2 min RetNet(8 x 5,1,25) to 28 h RetNet(8x5,1,300) for the on-premise execution at HPI, and from 1 s for RetNet(8x5,1,25) to 24 h for RetNet(8x5,1,300) when run as containerized at HPI, and from 4 s till 11 h for the containerized AWS execution.

The AWS execution is over two times faster than the two alternatives at HPI. This pattern is confirmed by the speed of Docker image builds. For the five Docker image builds, the average *NSP_Genesis* container build time was only 5.3 min at AWS, whereas the same build at HPI took 12.40 min. Furthermore, we notices a two-fold difference, which is surprising, assuming a “similar” simulation setting.

The memory utilization (as measured by RAM consumed) varies significantly from 19 MB for RetNet(8 × 5,1,25) to 16 GB for RetNet(8 × 5,1,300) execution on-premise at HPI, from 26 MB for RetNet(8 × 5,1,25) to 4.6 GB for RetNet(8 × 5,1,300) executing through a container at HPI, and from 68 MB to 17 GB for the containerized AWS execution. In the case of memory consumption, we notice that the on-premise (direct) HPI execution is similar to the containerized execution at AWS. Surprisingly, that consumption for the containerized HPI execution is four times smaller than in the other execution environments. On the contrary, the memory utilization for the smaller models (so with a neural column depth of 50, 75, and 100) that executed on-premise without the container at HPI is four times smaller if compared with their containerized execution at HPI.

We have also compared the standard and containerized simulation setup on the same underlying hardware. The results measured on the HPI on-premise infrastructure do not indicate any major negative impacts of containerization on the overall simulation performance. The average time (CPU time) needed to complete the containerized simulations of our RetNet models is 96.15% of the average simulation time needed to complete the same simulation on the virtual machine. Interestingly, the opposite was measured for memory consumption, and the containerized simulation consumed only 292% of the memory needed for a standard execution. The performance overhead of containerized execution is invisible so running computationally intensive neural simulations seem even more appealing, especially assuming the scalability and affordability of public cloud execution environments (Hale et al., 2017).

**Figure 7:**
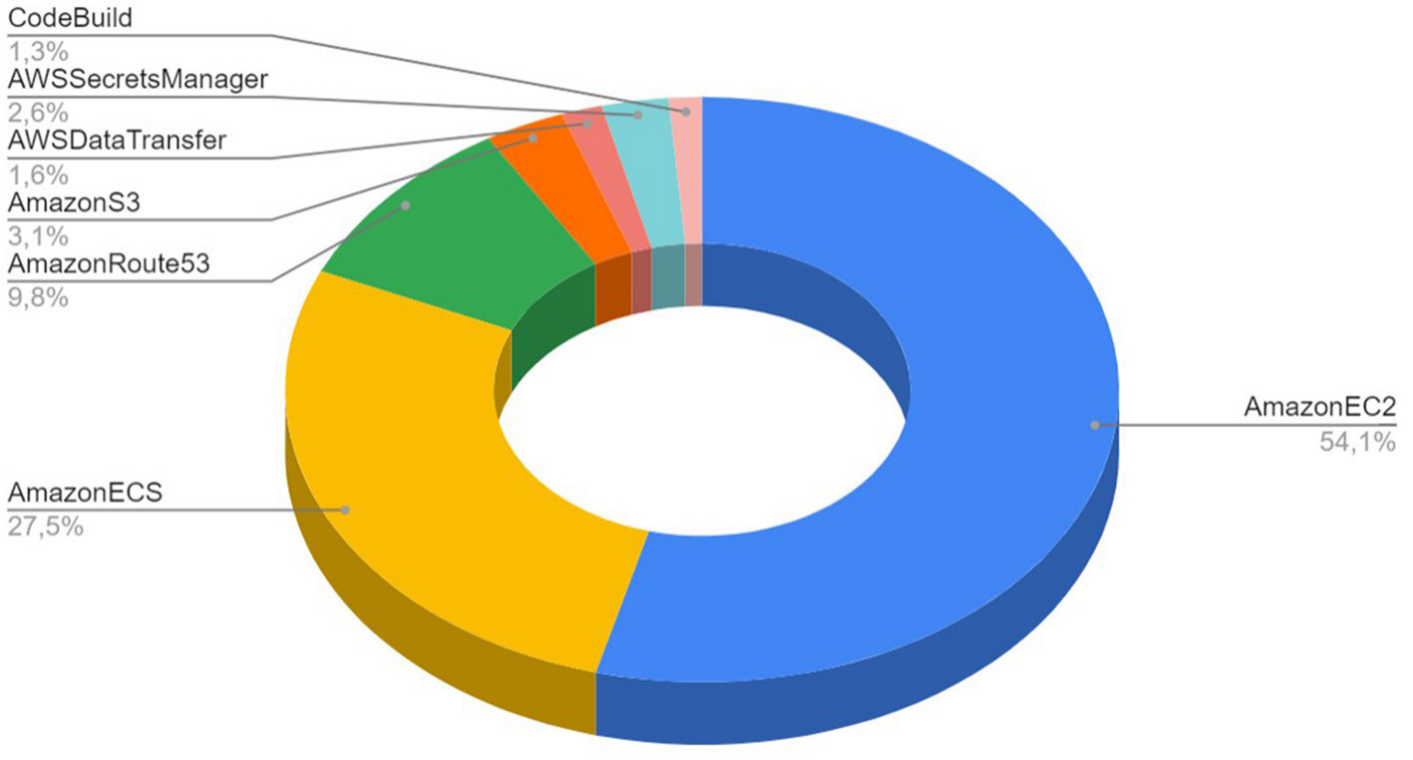
Cost structure for 18 simulations run using Neural Simulation Pipeline on AWS public cloud infrastructure.

The execution of simulations with NSP in AWS public cloud environment allowed us to investigate the cost per simulation, as well as the overall cost structure for the RetNet models. The overall cost structure is presented in Figure 7. We measured that 81.6% of the total cost is spent on AWS compute services (AWS ECS and Amazon EC2 spot instances). The rest of the cost is attributed to non-computational services: 3.1% on data storage (Amazon S3), 9.8% on Domain Name System (AmazonRoute53), 1.6% on data transfer, secure connection to GitHub 2.6% (AWS Secrets Manager), and 1.3% on automation (CodeBuild).

We have also calculated the real cost of each simulation. Simulating a single second of 1,040 HH neurons using RetNet(8 × 5,1,25) costs on average USD 0.02, while the most expensive RetNet(8 × 5,1,300) built with 12,040 HH neurons costs USD 4 to execute. A detailed cost per simulation is provided in Table 3.

## 4. Limitations and future perspectives

The current NSP requires a basic knowledge of the computer operating systems and the ability to run Bash scripts (and working knowledge of some AWS cloud services). In future, we would like to create a web application providing a simulation service using NSP containers without the need for running any scripts. This would allow us to expose NSP as a Scientific Workflow Management System to a wider community, gather feedback, and potentially also allow us to perform more extensive testing with (other than AWS) public cloud services providers.

That could lead to enhancing planning and forecasting of costs for large-scale simulations across different public clouds. In the current version, we have only used the AWS cost reports, as a source of cost information. We imagine that a trial run in a public cloud could help computational neuroscience researchers with their cost estimation. An automated trial run of a smaller model could be a good proxy for a full scale execution, and it would allow both easier and more accurate budgeting apart from just providing the researchers with simulation management and execution capabilities.

There are also a few other limitations in the current version of the neural simulation pipeline. First, the current version of our official NSP Docker image with the last version of GENESIS simulation engines is relatively large. It requires 1.17 GB in the local repository and 424.83 MB in the remote registry, that is after compression, at DockerHub.^8^ We believe that the image could be optimized by removing some uncritical operating system tools and utilities.

Second, the current neural simulation pipeline supports GENESIS and PGENESIS (Bower et al., 2003) only. We would like to create a version of the pipeline for each major (Tikidji-Hamburyan et al., 2017) simulation engine like BRIAN (Goodman and Brette, 2008), NEST (Gewaltig and Diesmann, 2007), and NEURON (Hines and Carnevale, 2001)/CoreNEURON (Kumbhar et al., 2019), as well as other (e.g., functional) simulators like Nengo (Bekolay et al., 2014). This will require a preparation of new *Dockerfile* in the *nsp-code* repository.

Looking at these plans, we recognize that some simulators may suit better for running in containers than the others (de Bayser and Cerqueira, 2017). We think that the next simulator to consider for NSP is NEURON (Hines and Carnevale, 2001)/CoreNEURON (Kumbhar et al., 2019). It is the most popular software for brain network simulations if counting the number of entries in ModelDB (Hines et al., 2004). Moreover, NEURON's architecture and installation^9^ resembles that of GENESIS, with the simulation setup requiring additional MPI libraries for parallel simulation. The next in line would be NEST,^10^ slightly less popular, but capable of running thread-parallel simulations “out-of-the-box” on multiprocessor computers with OpenMP (Dagum and Menon, 1998). For NEST, the application of NSP could benefit scientists, who would want to execute distributed simulations using MPI libraries. Finally, although the BRIAN software has monolithic architecture, it does not use external modules, or libraries, and it also does not use MPI parallelization. The benefit of using NSP could then be in enabling this software to run simulations in parallel on multiple nodes through the mechanism of NSP queues.

Third, at present, all the NSP containers are configured to read the file with simulation tasks from the Amazon S3 bucket at different moments in time. Nevertheless, a few containers could theoretically fetch the same simulation if they hit the file at the same moment in time. In future, we want to implement a proper semaphore mechanism for allowing or disallowing access to the simulation task. This problem could potentially also be resolved using Amazon SQS, a standard or first-in-first-out (FIFO) queue. Moreover, the NSP proof of concept was only tested with three containers reading the remote queue and executing the simulations in parallel. More containers could be evaluated to report detailed performance of the solution.

Fourth, we would like to facilitate the use of the ModelDB (Migliore et al., 2003) rather than the nsp-model GitHub repository so that inserting a new model into NSP could happen directly from ModelDB in a standard way (Hines et al., 2004).

Finally, we would like to redesign the NSP to provide a service for multiple research teams at the same time and enable interdisciplinary work between different profiles of researchers. That would likely require a web-interface for simulation management (mentioned already), as well as the security model based on a defined set of access rules, e.g., for model developers and/or neuro-scientists.

To summarize, the practical significance of NSP would be in reducing entry barriers to numerical systems modeling and large-scale simulations through a Docker-based pipeline, that could be executed across multiple compute infrastructures.

## 5. Conclusion

NSP provides a set of tools for automating the build of GENESIS and PGENESIS from its source code to container images. The simulation engines are bundled with all the necessary software libraries and allow for flexible testing and deployment of simulation code (e.g., of cybernetic simulation models) according to the IaC principle.

NSP tools also facilitate the analysis of experimental data. All simulation results are stored centrally, and available in a single, online storage. The experimental data are partially prepossessed, which facilitates further analysis by aggregating results and enriching them with additional information on the details of the execution environment and run-time statistics (e.g., runtimes, detailed information on processors, memory, and operating system processes).

We evaluated NSP using the liquid state machine RetNet models of up to 12,040 neurons, executed through GENESIS. We show how the containerized Docker-based pipeline designed by the authors allows the simulations to be developed, tested, and simulated in either an on-premise environment or in the public cloud environment. Finally, we describe the application of a novel simulation management method that simplifies model development and simulation across multiple execution environments, and we integrate the application of this method into the neural simulation pipeline.

We measured no overhead of containerization on CPU time for our RetNet model. The containerized execution was actually faster, taking only 96.15% of the average simulation time needed to complete the same simulation on the virtual machine. Interestingly, the opposite was measured for memory consumption, and the containerized simulation consumed only 292% of the memory needed for a standard execution. The performance overhead of containerized execution is invisible.

The simulation of our biological visual system was built of 12,040 HH neurons, that was executed for 11.62 h for US$ 4 only. The other finding was that only 81.6% of the total cost spent on AWS compute services is actually spent on AWS ECS and Amazon EC2 spot instances.

The practical significance of NSP is in reducing entry barriers to numerical system modeling and large-scale simulations, with application to both brain networks (GENESIS) and brain bio-mechanics (Kinetikit) simulations. The framework could also be used to improve experiment budgeting. NSP hides the complicated technical aspects of installing a simulation engine on different platforms, enabling the same model to be easily run on different types of processors and in-cloud computing with predefined service parameters. With this system and its functionalities, the developers want to popularize the use of computer simulators for brain research.

## Data availability statement

The original contributions presented in the study are included in the article/Supplementary material, further inquiries can be directed to the corresponding author.

## Author contributions

KC contributed to overall conceptualization, led the algorithmic development, simulation execution, data analysis, investigation, validation, and writing of the original draft. PS contributed to the algorithmic development, simulation execution, and data analysis. IK contributed to idea conceptualization. GW supervised the entire study. All authors participated in manuscript revision and approval of the submission.

## Footnotes

^1^List of Amazon Web Services. Available online at: https://aws.amazon.com/products/.

^2^Official DockerHub NSP Image. Available online at: https://hub.docker.com/r/karolchlasta/genesis-sim/tags.

^3^GENESIS showstat routine. Available online at: http://genesis-sim.org/GENESIS/Hyperdoc/Manual-25.html#showstat.

^4^NSP Code Repository, containing a source code of the pipeline. Available online at: https://github.com/KarolChlasta/nsp-code.git.

^5^NSP Model Repository, containing a source code of the simulations. Available online at: https://github.com/KarolChlasta/nsp-model.git.

^6^Official DockerHub Linux Image. Available online at: https://hub.docker.com//ubuntu18.04.

^7^Official DockerHub NSP Image. Available online at: https://hub.docker.com/r/karolchlasta/genesis-sim/tags.

^8^Official Docker Image Distribution Registry. Available online at: https://hub.docker.com/r/karolchlasta/genesis-sim.

^9^NEURON Documentation. Available online at: https://nrn.readthedocs.io/en/8.2.2/install/install_instructions.html.

^10^NEST Guide for Parallel Computing. Available online at: https://nest-simulator.readthedocs.io/en/stable/guides/parallel_computing.html.
